# Epilepsy Cured by Trephining

**Published:** 1830-04-01

**Authors:** 


### Epilepsy cured by Trephining.

Professor Dudley published some cases of this kind, in the first volume of the Transylvania Journal, and some cases are to be found in our own periodicals. The following is the most recent we have seen. The operation was performed by Dr. Guild, of Alabama, and the case is detailed in a late number of the American Journal of the Medical Sciences.

The patient was Capt. Elliott, aged 40 years, who had enjoyed good health till August, 1827, when he was suddenly attacked with an epileptic convulsion, threatening immediate death. Large bleedings saved him, but epilepsy at intervals of one, two, or three weeks, accompanied by racking pains in one side of the head, and entire loss of sight of the corresponding eye ensued. Various medicines were used in vain, and salivation was raised without effect. The fits became more frequent, and his life was in imminent danger. The trephine was proposed and consented to. The operation caused dreadful pain, and when the diploe was reached a profuse hæmorrhage ensued—that, however, subsided, and the bone was removed. It proved to be "rather of a carious and spongy nature, and somewhat thickened." The dura mater was healthy, and the brain pulsated, with the usual vigour as in health. As soon as suppuration was established, the health of the patient began to improve—the convulsions ceased—and the headache did not return. In thirty days the wound was cicatrized, and he has since enjoyed good health. Dr. Guild assumes it as proved that the cranium was the seat of the disease, "being thickened and thereby producing morbid irritation, congestion and epilepsy." But what produced the blindness of one eye?—and why has not Dr. G. told us what became of this part of the complaint. We have often to lament the loose and inaccurate manner in which some medical men detail their cases, and the hasty conclusions which they draw from inadequate premises.

If the bone was the seat of the disease, is it likely that the trephine took the whole of it away? For our own parts, we are disposed to think that the cause both of the epilepsy and the blindness was in the brain, and that the trephine cured one of them, at least in the way that a seton sometimes does—by inducing a large purulent discharge. It is just possible that a less hazardous wound kept in a state of copious suppuration might have led to the same successful result.

---

